# Integrating Immunologic Signaling Networks: The JAK/STAT Pathway in Colitis and Colitis-Associated Cancer

**DOI:** 10.3390/vaccines4010005

**Published:** 2016-02-29

**Authors:** Sebastian Zundler, Markus F. Neurath

**Affiliations:** Department of Medicine 1, University of Erlangen-Nuremberg, Kussmaul Campus for Medical Research and Translational Research Center, 91054 Erlangen, Germany; sebastian.zundler@uk-erlangen.de

**Keywords:** inflammatory bowel disease, colitis-associated cancer, cytokines, janus kinase, signal transducer and activator of transcription, IL-6

## Abstract

Cytokines are believed to be crucial mediators of chronic intestinal inflammation in inflammatory bowel diseases (IBD) such as Crohn’s disease (CD) and ulcerative colitis (UC). Many of these cytokines trigger cellular effects and functions through signaling via janus kinase (JAK) and signal transducer and activator of transcription (STAT) molecules. In this way, JAK/STAT signaling controls important events like cell differentiation, secretion of cytokines or proliferation and apoptosis in IBD in both adaptive and innate immune cells. Moreover, JAK/STAT signaling, especially via the IL-6/STAT3 axis, is believed to be involved in the transition of inflammatory lesions to tumors leading to colitis-associated cancer (CAC). In this review, we will introduce the main cellular players and cytokines that contribute to pathogenesis of IBD by JAK/STAT signaling, and will highlight the integrative function that JAK/STATs exert in this context as well as their divergent role in different cells and processes. Moreover, we will explain current concepts of the implication of JAK/STAT signaling in CAC and finally discuss present and future therapies for IBD that interfere with JAK/STAT signaling.

## 1. Introduction

Inflammatory bowel diseases (IBD) are characterized by chronic or relapsing inflammation of the intestine leading to symptoms including diarrhea, pain or hematochezia. Crohn’s disease (CD) and ulcerative colitis (UC) are considered to be the main entities of human IBD [1,2]. Although both diseases share similar clinical features and often manifest in younger patients, they considerably differ in several aspects. For example, UC is almost exclusively limited to the colon and is characterized by a continuous inflammation of superficial layers of the intestinal wall, while CD is marked by discontinuous and transmural inflammation that can be found throughout the gastrointestinal tract [3]. Several environmental factors affecting the risk for IBD development have been identified. Among these are smoking, appendectomy, use of antibiotics and lifestyle [4].

In spite of considerable advances, however, the pathogenesis of IBD has still not been fully elucidated. Nevertheless, several pathogenetic pillars have been identified that crucially contribute to development or propagation of inflammation. As a result, IBDs are generally thought to occur in genetically predisposed patients with a weakened intestinal barrier and a reduced biodiversity of the enteric microbiota [5,6,7]. Subsequently, epithelial translocation of noxious antigens to the lamina propria leads to a dysregulated and undercontrolled immune response maintaining itself and leading to damage of the intestinal wall [3].

The importance of genetic factors has been extensively studied and large numbers of genes have been identified that are associated with either CD, UC or both. Among these are several genes encoding or regulating cytokines and receptors that signal via the janus kinase (JAK)/signal transducer and activator of transcription (STAT) pathway, e.g., IL-23R, IL-10, IFN-γ or IL-12B [5]. Moreover, single nucleotide polymorphisms in loci that contain the genes of Jak2, Tyk2, STAT1, STAT3 and STAT4 as direct constituents of the JAK/STAT signaling cascade have been reported to entail an increased risk for the development of IBD [5,8].

Weakness of the epithelial barrier in IBD is a consequence of a disequilibrium of protective and aggressive factors. For example, protective mechanisms like mucin secretion, production of defensins or intestinal epithelial restitution of small wounds are impaired [9,10,11]. Again, an involvement of JAK/STAT signaling in these processes has been postulated [12]. Moreover, a link from the intestinal microbiota to JAK/STAT signaling has been proposed [13,14]. And finally, the misguided immune response in IBD crucially depends on JAK/STAT, e.g., in terms of differentiation and proliferation of important immunologic players [3].

Chronic intestinal inflammation in IBD potentially leads to the development of colitis-associated cancer (CAC). In the course of inflammation, the epithelial turnover is high, mutagens may easily cross the defective mucosal barrier, oncogenic signaling cascades are activated, and reactive oxygen species are produced. This leads to progressive accumulation of genetic and epigenetic mutations manifesting stepwise first as low- and high-grade dysplasia and later as colorectal carcinoma [15]. This affects up to 18% of patients with longstanding colitis and is a major cause of mortality in IBD patients [16]. Several mediators that are critically involved in IBD pathogenesis have also been shown to be implicated in the process of malignant transformation [17] and, moreover, IBD and CAC are linked by cytokine profiles that resemble each other in many aspects (e.g., elevation of IL-1, IL-6, IL-23, TNF) [15]. Hence, a close and causal relationship between colitis and CAC is well established both clinically and on a molecular basis. Concerning the latter, especially IL-6 has emerged as main mediator of inflammation-associated tumorigenesis, and its signaling essentially depends on STAT3 [18]. Thus, JAK/STAT molecules are also implicated in neoplastic transformation in long-standing colitis.

After outlining the principles of JAK/STAT signaling, this review will discuss the present evidence for the involvement of JAK/STAT pathways in the pathogenesis of IBD and CAC preceded by a short summary of JAK functions and subsequently arranged according to the different members of the STAT family. The functions of the different STATs in the cellular compartments of lymphocytes, macrophages and intestinal epithelial cells (IECs) in IBD are depicted in Table 1. Finally, we will summarize possible implications of these findings for clinical therapy.

## 2. Principles of Janus Kinase (JAK)/Signal Transducer and Activator of Transcription (STAT) Signaling

**Figure 1:**
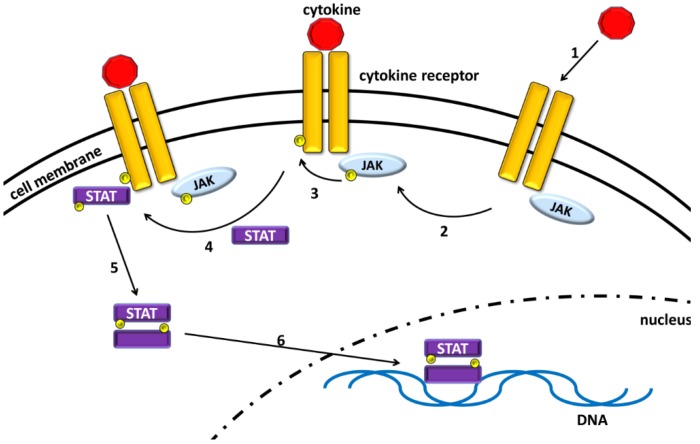
Principles of JAK/STAT signaling. Upon binding of a cytokine to its receptor (1) conformational changes lead to JAK auto- or transphosphorylation (2) and subsequent phosphorylation of the receptor by JAKs (3). This creates binding sites for STAT molecules, which are also phosphorylated by JAKs (4). Phosphorylated STATs dimerize (5) and translocate to the nucleus, where they control transcription by directly binding to the DNA (6).

JAK/STATs translate the signal of a large number of cytokines and interferons from the cell membrane to the nucleus. Upon binding of a particular ligand to its respective transmembrane receptor, receptors dimerize and lead to conformational changes in associated JAK molecules [19] (Figure 1). This confers catalytic activity to the JAKs resulting in first auto- or transphosphorylation of the JAKs and second phosphorylation of the cytokine receptors by these phosphorylated JAKs [20]. The phosphorylated sites of the cytokine receptor now serve as docking regions for STATs, which are also transphosphorylated by JAKs [21]. Thereupon, STATs are able to homodimerize and translocate to the nucleus, where they directly bind to the DNA, thereby regulating the transcription of a wide number of genes [20].

As there exist four different JAK molecules (JAK1, 2, 3, TYK2) and seven members of the STAT family (STAT1, 2, 3, 4, 5a, 5b, 6), specific combinations of a cytokine receptor and the activated JAKs and STATs can lead to specific cellular responses [22]. However, there is a significant overlap between the JAK/STAT signaling of different cytokines or interferons, and most receptors are linked to more than one STAT molecule. Thus, how a distinct pattern of gene activation or repression is induced by each messenger is still a matter of debate [23]. Globally, there are sets of related receptors that induce activation of the same set of JAK and STAT molecules. These are the interferon family receptors (e.g., for IFN-α, -β, -γ, IL-10, IL-22), the gp130 family receptors (e.g., for IL-6, IL-11, IL-12, IL-23), the γC family receptors (e.g., for IL-2, IL-4 IL-7, IL-9, IL-21), the IL-3 family receptors (for IL-3, IL-5, GM-CSF) and the single chain family receptors (for hormones such as growth hormone or erythropoietin) [24].

It must nevertheless be mentioned that signaling via JAKs and STATs is not always as straightforward as outlined above. For example, JAK/STAT signaling may differ between different members of these families. Moreover, “non-canonical” pathways in which STATs are activated by other upstream signaling molecules [25,26], or JAKs phospohorylate other downstream targets [27], have also been shown. Furthermore, additional phosphorylation or modification of STATs can mediate their activity, and functions for STATs in the mitochondrium have also been demonstrated [22]. However, the pathway of JAK/STAT signaling depicted above is deemed the most important, and has garnered the most attention in the context of IBD and CAC.

### 2.1. JAKs

As STATs are the final effectors of JAK/STAT signaling, and each JAK is related to several STATs, most research on this signaling pathway in IBD has focused on STATs. However, there are some basic conclusions that can be drawn from reports with a general immunologic background and the limited number of studies directly assessing JAKs in colitis.

JAK1- and JAK2-deficient mice die pre- or perinatally [28,29,30]. JAK3 knockout mice show defective signaling of IL-2, IL-4, IL-7, IL-9 and IL-15 and suffer from severe combined immunodeficiency, as do patients with genetic defects in JAK3 [31,32]. Finally, Tyk2 deficiency in mice is associated with defective IFN-α, IL-12 and IL-18 signaling leading to perturbation of T and B cell development [33,34,35]. This demonstrates that all these JAK molecules are essential for a normal function of the whole organism and suggests that their implication in the aberrant immunologic response in colitis and CAC follows the general rules of JAK/STAT signaling.

A recent study directly addressed the function of Tyk2 in experimental colitis using Tyk2 knockout mice. Such mice displayed severer experimental colitis, reduced phospho-STAT3 levels and decreased proliferation of IECs. This could be confirmed in mice with a specific deletion of Tyk2 in IECs and linked to defective IL-22 signaling [36]. However, it is in contrast to another report that demonstrated reduced experimental colitis in Tyk2^−/−^ mice and suggested reduced Th1 and Th17 activity to be responsible for this observation [37].

In JAK3 knockout mice, a spontaneous onset of IBD-like symptoms has been reported and was connected to changes in macrophage and lymphocyte populations [38]. Later, defective intestinal barrier function was suggested to contribute to colitis in JAK3-deficient mice as well [39]. Unfortunately, other studies with genetically engineered mice (e.g., conditional knockout mice) directly assessing the function of JAKs in the intestinal compartment are lacking so far. Thus, reliably assessing the functional role of JAKs in IBD and CAC is difficult, especially as a pharmacologic approach to assess this question is limited by the fact that most of the available JAK inhibitors are not completely selective and target several members of the family. An exception is a study testing a specific JAK2 inhibitor in a murine CAC model showing that this compound inhibited *de novo* neoplasia and led to regression of established tumors, which correlated with STAT3 activation [40]. Taken together, however, further insights into the specific function of JAKs in colitis and CAC are highly desirable.

### 2.2. STAT1

**Figure 2:**
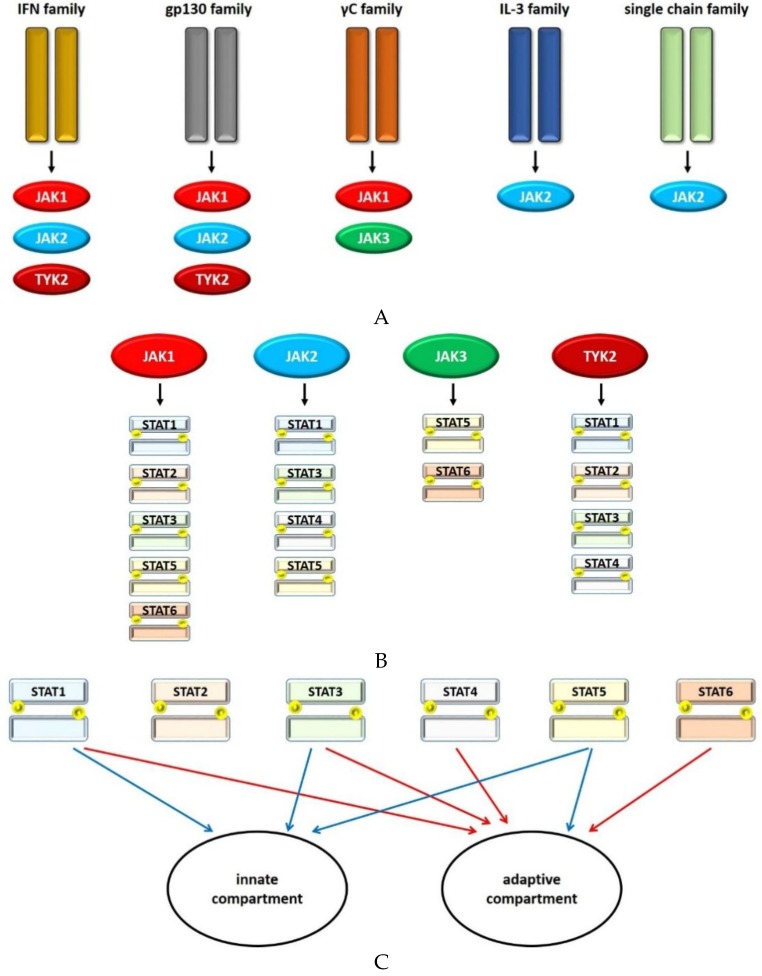
Schematic illustration of the signaling network of JAK/STATs in IBD. (A) Different receptor families activate distinct sets of JAK molecules. (B) JAK molecules, in turn, phosphorylate assorted STATs. (C) Based on present evidence, these seem to exert either pro-inflammatory (red arrows) or protective (blue arrows) functions concerning the pathogenesis of IBD in adaptive and innate immunity.

STAT1 is phosphorylated by either JAK1 and JAK2 or JAK1 and TYK2 and is thought to be of special relevance in the context of signaling via the IFN-γ receptor and the related family of receptors (Figure 2). However, it may also be activated by signaling originating from members of the gp130 and γC family of receptors [24]. IFN-γ is an important effector cytokine of Th1 cells, which have been associated with CD pathogenesis [41].

A first study of STAT1 expression in IBD reported an increase of STAT1 levels in gut tissue from both UC and CD. Phosphorylated, *i.e*., activated STAT1 was especially upregulated in monocytes and granulocytes [42]. Another investigation revealed higher levels of total but not phospho-STAT1 in CD but not UC compared with controls [43]. Similarly, STAT1 gene expression was elevated in CD patients in a genome-wide gene expression study [44].

Different suggestions can be made as to how this might be functionally relevant. While an amelioration of experimental colitis was reported in an initial assessment with STAT1 knockout mice [45] and upon specific pharmacologic inhibition of STAT1 in T cells [46], it has also been shown that reduced IL-19-dependent STAT1 signaling in macrophages results in enhanced production of pro-inflammatory cytokines [47]. On the other hand, repression of STAT1-mediated gene transcription by suppressor of cytokine signaling (SOCS)1 is essential to ensure the suppressive function of regulatory T cells (Tregs) by inhibiting IFN-γ production [48], and induction of Th1 development through IL-27 depends on STAT1-mediated suppression of Th2-driving GATA3 [49]. On the contrary, in intestinal epithelial cells (IECs), the activation of indoleamin 2,3-dioxygenase (IDO-1), an enzyme with antibacterial and anti-inflammatory properties, also relies on STAT1-activation by IL-27 [50]. Conversely, some evidence suggests that several molecules with anti-inflammatory properties cause a decreased STAT1 phosphorylation in lymphocytes [51,52]. Moreover, the bacterial metabolite butyrate inhibits IFN-γ-induced JAK2 activation and subsequent STAT1 signaling [13], which could be interpreted as a potential mechanism of cross-talk between the enteric microbiome and the intestinal immune system.

Taken together, the precise role of STAT1 in IBD remains unclear as it is implicated in both pro- and anti-inflammatory pathways. Thus, the upregulation of STAT1 expression in human IBD could be seen either as a primary defect contributing to the development of intestinal inflammation or as the secondary attempt of the immune system to limit inflammation. However, it appears that STAT1 activation in lymphocytes rather supports IBD development, while its phosphorylation in macrophages and epithelial cells seem beneficial regarding intestinal inflammation. Variations on this theme of differential roles in adaptive and innate cells will reappear several times in the further course of the portrayal of JAK/STAT signaling in IBD.

STAT1 has also been proposed as important in CAC. Here, it seems to have a protective function as normal intestinal fibroblasts, but not activated fibroblasts or fibroblasts from patients with IBD, activate STAT1 in colon cancer cells and thereby inhibit the growth of the tumor cells [53]. Further studies directly addressing CAC have not yet been undertaken, but some deductions can be made from findings relating to colorectal cancer (CRC) in general as these findings involve molecules that also regulate inflammation. For example, the frequent KRAS mutation in CRC cells leads to inactivation of STAT1 and subsequent insensitivity of tumor cells to the anti-tumorigenic effects of IFN-γ [54]. IFN-γ, in turn, induces apoptosis of tumor cells through a STAT1-dependent pathway [55]. This is in contrast to findings in a mouse model, where STAT1 activation was critical for the spontaneous development of CRC in mice deficient in SOCS1, probably through inducing enzymes leading to oxidative stress [56]. The connection linking STAT1 activation with the production of reactive oxygen species has also been established in murine peritoneal macrophages *in vitro* [57]. In another murine model of colon cancer, however, intestinal polyp formation was equal in animals carrying a STAT1 deletion compared with STAT1-expressing mice [58], and SOCS1 has also been proposed as an oncogene mediating its effect through STAT1-downregulation [59]. In conclusion, the role of STAT1 for cancer development remains controversial and requires further study.

### 2.3. STAT2

STAT2 is predominantly involved in the signal transduction of type I interferons (*i.e.,* IFN-α and β) [60]. As these interferons are mainly thought to engage in the response to viral infections, STAT2 has only barely been investigated in the context of IBD. It even seems to be downregulated in UC and CD [43], and no important involvement of STAT2 in intestinal inflammation has been uncovered to date.

However, IFN-α possesses anti-tumor activity [61] and some evidence suggests that STAT2 might be of relevance concerning cancer development. Tumor cells repress the transcription of certain genes by virtue of DNA methyltransferases. In experiments with inhibitors of DNA methyltransferase, it could be demonstrated that STAT2 was upregulated in response to such treatment, going along with the ability of IFN-α2a to inhibit cell growth [62]. Moreover, activating mutations of KRAS as they occur in colon cancer downregulate STAT2 expression, which might contribute to the reduced sensitivity of tumor cells to interferons [54].

### 2.4. STAT3

In contrast to STAT2, the essential role of STAT3 in IBD is beyond question. STAT3 signaling is a hallmark of many members of the gp130 family of cytokines (e.g., IL-6, IL-11), but some IFN family members like IL-10 or IL-22 also require STAT3 to exert their functions. JAK1 together with JAK2 or TYK2 activates STAT3 [24].

In both UC and CD lamina propria T cells, STAT3 expression and nuclear translocation is induced together with downstream anti-apoptotic genes. This finding was attributed to IL-6 signaling as blockade of IL-6 trans signaling enhanced apoptosis of T cells in experimental colitis [63]. Consistently, other studies also reported elevated expression of STAT-3 and/or phospho-STAT-3 in IBD [43,44,64]. Moreover, hyperactivation of STAT3 in mice missing SOCS3 activity led to severer colitis [65]. Compatibly, treatment of mice with STAT3 antisense oligonucleotide ameliorated experimental colitis and induced apoptosis of lamina propria mononuclear cells (LPMCs) [66]. Similarly, growth hormone caused a downregulation of STAT3 signaling which led to less severe murine colitis going along with increased LPMC apoptosis. Of note, proliferation and apoptosis of epithelial cells were increased and reduced, respectively, [67] establishing a connection to the below remarks on STAT3 in CAC.

Later, another mechanism of STAT3-mediated pathogenic contribution was detected as STAT3 was found to be essential for the differentiation of Th17 cells and Th17-dependent murine colitis [48,68,69]. Th17 development depends on an environment of IL-23-, IL-6-, IL-21-, IL-1β- and TGF-β-signaling and includes transcriptional control by STAT3 [70]. The pathogenetic relevance of Th17 cells in IBD has been proven by genetic suppression of several genes responsible for Th17 lineage commitment that results in decreased severity of experimental colitis [71,72,73]. Thus, STAT3 seems to critically regulate not only T cell survival in response to IL-6 but also controls the differentiation of a CD4+ T cell population with high relevance for the development of both UC and CD.

Interestingly, however, several cytokines that have anti-inflammatory properties also make use of the STAT3 pathway. In concrete terms, IL-22 has been shown to induce epithelial STAT3 activation and reduce disease activity of experimental colitis in mice via recovery of goblet cells and restoring the mucus layer [74]. This was affirmed by a study with specific disruption of STAT3 in epithelial cells, which showed that IL-22-dependent STAT3 activation is indispensable for intestinal epithelial restitution [12]. Similarly, IL-24 activated STAT3 in epithelial cells and promoted mucin expression [75] and toll like receptor 2-mediated IL-11 secretion resulted in epithelial STAT3 activation and maintenance of mucosal integrity [76]. What seems contradictory to the deleterious functions of STAT3 depicted above could be explained by the fact that STAT3 activation was investigated in different cellular compartments, *i.e.*, lymphocytes and epithelial cells. Thus, different functions of STAT3 may be due to cell-specific signaling. The general notion is that while STAT3 controls pro-inflammatory functions in cells of the adaptive immune system, its role in innate immune cells is rather protective [77]. This perception is also based on the observation that inactivation of STAT3 in neutrophils and macrophages causes chronic murine colitis via increased Th1 responses and decreased signal transmission of IL-10 [78]. Another study using a model with STAT3 deletion predominantly occurring in macrophages and IECs confirmed these results [79]. Moreover, disruption of STAT3 in bone marrow cells resulted in a CD-like phenotype in mice prior to death [80], and suppression of experimental colitis by CX3C chemokine receptor 1-bearing myeloid cells was dependent on STAT3 activation by IL-10 [81]. Conversely, STAT3-induced expression of IL-10 was responsible for the protective effects of epithelial CD1d in regard to intestinal inflammation [82].

However, this view of divergent functions of STAT3 in different immunologic compartments is not fully conclusive as an important role of STAT3 has also been shown for the IL-10-dependent suppressive function of regulatory T cells. Regardless of the diverse features of STAT3 in the pathogenesis of IBD, however, all these data perfectly match with the aforementioned association of mutations in the STAT3 locus with increased risk for both CD and UC [83].

In addition to these important effects in human and murine IBD, there is growing evidence that STAT3 signaling might be an important link connecting IBD and CAC. Paralleling the previously mentioned findings in IECs, T cell- and macrophage-derived IL-6 enhanced tumor cell growth in murine colorectal cancer [84]. This was related to CAC by the observation that STAT3 hyperactivation in IECs not only led to decreased susceptibility for colitis but also to increased occurrence and growth of tumors through IL-6 and IL-11 [85]. Consistently, IL-6 signaling via STAT3 was demonstrated to crucially mediate protection from apoptosis and induction of proliferation in IECs [86]. Thus, the immunologic signaling network in IBD could directly trigger neoplastic transformation in an attempt to limit intestinal inflammation.

Interestingly, a non-canonical function of STAT3 in mitochondria has been proposed to mediate STAT3-dependent resistance of colorectal cancer cells to apoptosis by decreased production of reactive oxygen species [87]. Furthermore, a direct link between colitis induction by enterotoxigenic *Bacteroides fragilis*, a commensal bacterium of the intestinal flora, and tumor formation via STAT3 could be established [88]. Additionally, a mechanism of STAT3 signaling in CAC originating from vascular endothelial growth factor receptor (VEGFR) has been described. VEGFR2 was activated on IECs from patients with CAC, and VEGFR signaling via STAT3 was required for proliferation of IECs and tumor growth in mice [89]. In subsequent studies, IL-21- and IL-22-dependent STAT3 activation [90,91] were also linked with the transition of chronic colitis to CAC.

As a consequence, it is not surprising that an upregulation of IL-6 and phospho-STAT3 has also been demonstrated in patients with dysplasia or cancer originating from UC [92]. Generally, based on the present evidence, the IL-6/STAT3 axis is considered the best characterized and most important mechanism of neoplastic transformation of chronic inflammatory lesions in the colon [18].

Collectively, STAT3 activation via the JAK/STAT pathway is of central importance in both IBD and CAC. Though not all findings can as of yet be easily reconciled, it can be conjectured that STAT3 acts in a rather pro-inflammatory manner in the adaptive immune compartment, while its functions in innate defense mechanisms are rather protective. Yet, the cost of the latter observation seems to be a concurrent increase of the risk of cancer development.

### 2.5. STAT4

JAK2/TYK2-dependent phosphorylation of STAT4 is virtually exclusively found in response to signaling of certain members of the gp130 family of receptors, especially IL-12R and IL-23R [24] that share a common subunit [93].

The postulated involvement of STAT4 in IBD is predominantly linked to the essential role of STAT4 for the development and function of Th1 cells. Based on an enhanced expression of both Th1-related transcription factors like T-bet and STAT4 and Th1-secreted cytokines like IFN-γ and IL-2, these cells are deemed critical for the pathogenesis of CD rather than UC [70]. IL-12 is a central cytokine governing Th1 lineage commitment and thus, IL-12-mediated STAT4 phosphorylation with subsequent IFN-γ secretion explains the tight association of STAT4 with Th1 cells. Similarly, STAT4 regulates IFN-γ expression in natural killer (NK) cells [93]. Moreover, IL-21-dependent STAT4 phosphorylation has been linked to Th1 activity [94].

In experimental models, deletion of STAT4 brought about a relative protection from colitis development [95] and, conversely, STAT4 hyperactivation resulted in spontaneous development of transmural colitis with a Th1-like immunologic profile, thus resembling human CD [96]. The isoform STAT4β seems to mediate more detrimental effects than STAT4α, which was explained by regulation of TNF-α and GM-CSF production [97]. Moreover, STAT4 has been postulated to contribute to suppression of regulatory T cells by Th1 cells in murine colitis [98]. Plasticity of T cell lineages has been increasingly acknowledged as of late and STAT4 was shown to be involved in the transition of Th17 cells to colitogenic Th1 cells [99].

In human IBD, an association of STAT4 polymorphisms and both UC and CD has been uncovered [5,100,101]. Furthermore, two studies reported an increased expression of both constitutive and active STAT4 in the mucosa of patients with UC [102,103]. This is surprising as Th2 rather than Th1 cells are considered as disease-driving in UC [41]. However, this might be explained by the fact that IL-23, which is crucial for the differentiation of Th17 cells, can also activate STAT4 [104], and Th17 cells have also been shown to be crucial for the pathogenesis of UC [105]. Consistent with the aforementioned experimental data on STAT4 isoforms, a recent study found an increase in the ratio of STAT4β and STAT4α in IBD patients, but not controls [106].

Only limited evidence is available concerning STAT4 in CAC. However, IL-12 has been demonstrated to be a cytokine with potent anti-tumor activity [93] and STAT4 signaling in NK cells has been shown to be involved in this effect [107]. A potential role of the STAT4 molecule in cancer has also been suggested based on an association of rectal cancer and genetic variations in STAT4 [108]. Consistently, STAT4 is overexpressed in samples from colonic carcinoma and its expression correlates with improved survival [109].

### 2.6. STAT5

STAT5 activation predominantly occurs in response to JAK1 and JAK3 activation by members of the γC family of receptors and JAK2 signaling originating from receptors of the IL-3 family and the single chain family [24].

Growth hormone belongs to the latter family and has anti-inflammatory functions in experimental colitis. Reduced growth hormone signaling has been linked to impaired STAT5b activation. Interestingly, the blockade of tumor necrosis factor (TNF)-α, which is successfully used in clinical therapy of patients with IBD, was demonstrated to re-induce signal transduction via this axis [110]. Similarly, chronic growth hormone administration is protective in experimental colitis through induction of STAT5b [111]. This is consistent with an increased susceptibility of STAT5b-deficient mice for chemically induced colitis [112]. Later, this was linked to enhanced apoptosis of epithelial cells and subsequent damage of the mucosal barrier [113].

Another protective mechanism for STAT5 in IBD also concerns the epithelium: STAT5 is critical for the proliferation of intestinal epithelial stem cells and subsequent regeneration of the crypt epithelium [114].

Apart from this, STAT5 is involved in the regulation of T cell differentiation like other STATs; it is especially important for Foxp3 induction in regulatory T (Treg) cells as deletion of STAT5 in T cells disrupted Treg differentiation in an IL-2Rβ-dependent manner [115]. In addition, IL-2 signaling via STAT5 limits Th17 in favor of Treg development [116], whereas STAT3, a main regulator of Th17 commitment, negatively controls Treg differentiation [117]. Under physiologic conditions, Tregs are essential for preventing autoimmunity by limiting immune responses. In experimental colitis, Tregs can suppress disease development [118] and in human IBD, increased Tregs are overwhelmed by the strong expansion of effector T (Teff) cells [118,119] and therefore not able to control inflammation.

Some interesting findings have also been reported for STAT5 signaling originating from granulocyte macrophage colony stimulating factor (GM-CSF), although the implications for colitis are not fully resolved. Wan *et al.* showed that while IL-21-dependent STAT3 activation increased apoptosis of dendritic cells (DCs), GM-CSF-induced STAT5 signaling offers protection from apoptosis [120]. Conversely, STAT5 was found to control the development of a novel CD4+ T cell subset producing GM-CSF that was crucial in inducing experimental autoimmune encephalitis, but not colitis [121].

Taken together, it appears that STAT5 has rather anti-inflammatory properties both in the epithelium and in adaptive immunity. To the contrary, the role of STAT5 in CAC is far from being understood and can only be deduced from the poor evidence that is available for colorectal carcinoma (CRC) in general. Here, phospho-STAT5 expression is associated with higher levels of cyclin D1 and poor prognosis [122], and single nucleotide polymorphisms in both STAT5a and STAT5b are linked with colon cancer [108]. Experimentally, STAT5 has been demonstrated to be involved in a CCL-2-dependent increase of vascular permeability, thus facilitating metastases [123]. Disruption of STAT5 signaling in human CRC cells led to cell cycle arrest, inhibited cell migration and invasion [124] and resulted in apoptosis of CRC cells. Of note, unphosphorylated STAT5 was proposed to act as a tumor suppressor by heterochromatin stabilization [125].

### 2.7. STAT6

STAT6 phosphorylation occurs upon JAK1 and JAK3 activation and is virtually only induced by signaling via some members of the γC family of receptors, namely IL-4R and IL-13R [24].

Besides GATA3, STAT6 is the most important transcription factor determining Th2 differentiation. Additionally, interplay of STAT6 with PU.1 and IRF4 is implicated in the generation of the recently described Th9 subset of CD4+ effector cells. Both lineages are thought to be pathogenic especially in UC [70].

Compatibly, STAT6-deficient mice are relatively protected from oxazolone-induced colitis, which is accompanied by a reduction of Th2 cytokines [126]. Moreover, STAT6 critically mediates the pro-inflammatory properties of the Th2 signature cytokine IL-4 in murine colitis [127], causes IL-4-dependent inhibition of Foxp3 and, thus, reduced Treg induction [128]. IL-13 as another key cytokine in UC was shown to induce apoptosis in IECs via STAT6 [129]. Again, though, STAT6 may be of differential relevance in the innate compartment of the immune system as STAT6-dependent IL-4 signaling is responsible for the polarization of M2-like macrophages, which support restoration of the mucosal barrier [130].

In human IBD, phospho-STAT6 is increased in UC [129,131], and a polymorphism of STAT6 was linked to a subgroup of CD patients [132]. Thus, the overall importance of STAT6 seems to be predominantly due to STAT6 signaling in the adaptive immune system.

Regarding CAC, Wick *et al*. reported a shift from preferential STAT6 activation to STAT3 activation regarding non-cancerous and cancerous samples from IBD patients [133], respectively, suggesting that STAT6 might prevent malignant transformation or might be downregulated in a neoplastic environment. *In vitro* evidence, however, rather favors the view that STAT6 supports carcinogenesis by promoting proliferation and preventing apoptosis of epithelial cells [134,135]. Consistently, IL-12-induced apoptosis of CRC stem cell was associated with reduced signaling via the IL-4/STAT6 axis [136]. Collectively, these data are only a preliminary glimpse on the nature of STAT6 in CAC and further studies are required for clarification.

## 3. Therapeutic Implications

**Figure 3:**
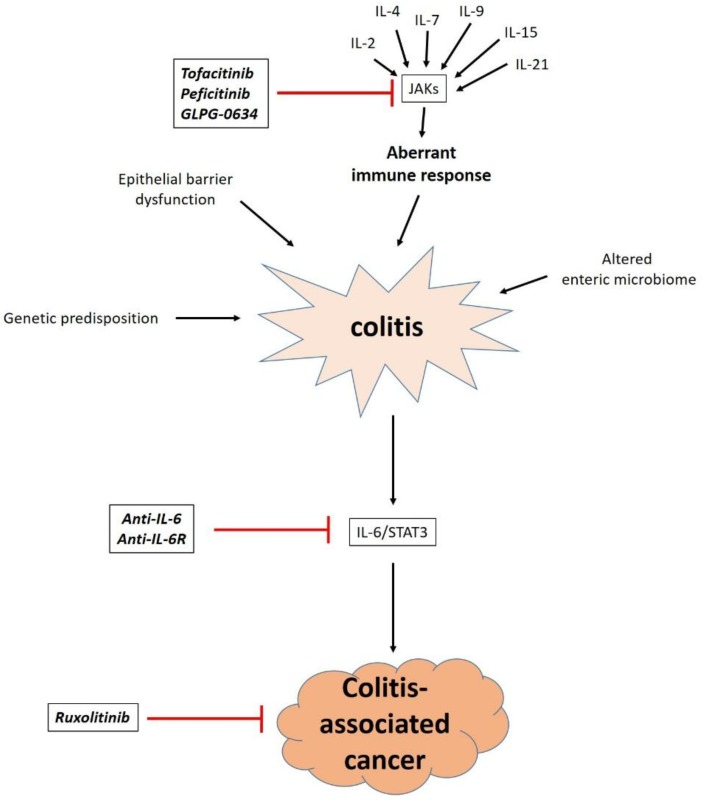
Schematic illustration of currently evaluated compounds interfering with JAK/STAT signaling in the context of colitis and CAC. An aberrant immune response essentially contributes to colitis and many of the responsible cytokines signal through JAKs, which are targeted by tofacitinib, perfecitinib and GLPG-0634. IL-6/STAT3 signaling is believed to lead to development of CAC, and inhibiting IL-6 or its receptor might therefore prevent malignant transformation. JAK inhibition by ruxolitinib might become an option in manifest CRC.

Although STAT molecules are the ultimate effectors of JAK/STAT signaling, currently tested therapeutic applications in IBD predominantly target JAKs, thus interfering with the signaling of a variety of cytokines (Figure 3). This is also the main difference to current therapies and other compounds under development. While conventional treatments unselectively suppress the immune system or the inflammation it causes [3], therapeutic anti-cytokine antibodies target one (e.g., infliximab) or a maximum of two (e.g., ustekinumab) cytokines [137,138]. On the contrary, JAK/STAT inhibition might represent a solution in between these two extremes as targeting the integrative function of these molecules allows selective inhibition of several cytokines at the same time. Another interesting aspect is that––unlike monoclonal antibodies that have to be given intravenously––therapeutic JAK/STAT inhibition through small molecules might be achieved through administration *per os* and therefore be more convenient for patients.

The compound that is furthest advanced in clinic trials is tofacitinib, which predominantly inhibits JAK1 and JAK3. In a phase II trial in patients with UC, up to 78% of the patients receiving tofacitinib had a clinical response after eight weeks compared with 42% in the placebo group. Remission was noted in up to 48% *vs*. 10%, and the side effect profile was judged tolerable [139]. Meanwhile, two phase III induction trials have been completed but not yet published, and a phase III maintenance study is ongoing. Conversely, tofacitinib was not superior to placebo in inducing a clinical response in patients with CD [140]. It could be reasoned, that this might be related to the predominant implication of JAK3 in the signaling of some cytokines from the γC family (IL-4, IL-9, and IL-13) that seem to be of special importance for UC.

Moreover, the JAK inhibitors peficitinib and GLPG-0634 are currently being tested in early-stage clinical trials [141].

What is also worth discussing in this context due to its close relation to JAK/STAT signaling and its probable implication in CAC development is anti-IL6R or anti-IL6 treatment. Regarding the central role of IL-6 in the orchestration of immunologic events in IBD, great hopes have been placed in blocking IL-6 signaling. However, data from clinical trials are thus far scarce [142], and adverse outcomes have been reported [143]. Yet, interfering with IL-6 and subsequent STAT3 signaling is still deemed promising especially as such therapy might reduce the risk of CAC [144].

Finally, JAK inhibition might also become an option for manifest CRC or CAC. In a mouse model of colitis-induced colorectal cancer, JAK2 inhibition resulted in reduction of proliferation and angiogenesis and down-sized established tumors [40]. As a consequence, the JAK inhibitor ruxolitinib, which has already shown promising results in the treatment of hematologic disorders [145], is currently being evaluated in metastatic colorectal carcinoma in clinical trials [141].

## 4. Conclusions

Clearly delineating the role of JAK/STAT signaling in IBD and CAC is difficult due to the pleotropic functions of the single molecules. However, some general conclusions concerning the pathogenesis of IBD can be made based on the above depictions. First, while most reports favor a rather aligned role of JAK/STATs for immunologic processes within the same immunologic compartment, opposing features of JAK/STAT signaling in innate and adaptive immunity are not uncommon. Second, the translation of findings from rodent models into clarification of the human situation is still in progress as most human data concern expression studies or genetic associations. This may be a direct consequence of differential JAK/STAT signaling in different cells or environments because this makes it difficult to draw straightforward conclusions from applying inhibitors of single molecules. Third, what is protective in JAK/STAT signaling in IBD seems to be rather disease-driving in CAC regarding several STATs. This is likely due to the fine line between regeneration and uncontrolled proliferation. However, detailed and coherent functional data regarding the implication of JAK/STAT signaling in CAC development are only available for STAT3. And fourth, although several obstacles are present as outlined, therapeutic application of compounds that interfere with JAK/STAT pathways are within reach. Hopefully, further advances in basic science will help to develop drugs that selectively target the detrimental consequences of JAK/STAT signaling, while clinical research will identify indications and patient groups in which interfering with JAK/STAT signaling is a promising treatment.

## Figures and Tables

**Table 1:** Summary of important STAT functions in different cellular compartments. For details, confer Section 2.2, Section 2.3, Section 2.4, Section 2.5, Section 2.6 and Section 2.7).

Cell type	STAT	Effects and functions in IBD
Lymphocytes	STAT1	Inhibition → amelioration of colitis Important for Th1 function, but impedes Th2 and Treg function
	STAT3	IL-6-mediated resistance to apoptosis Inhibition → amelioration of colitis drives Th17 differentiation promotes IL-10-dependent Treg function
	STAT4	drives Th1 differentiation, suppresses Treg function Deletion → protection from colitis hyperactivation → spontaneous colitis
	STAT5	drives Treg differentiation, limits Th17 differentiation
	STAT6	drives Th2 and Th9 differentiation, reduces Treg induction Deficiency → protection from colitis, less Th2 cytokines
Macrophages	STAT1	IL-19-dependent reduction of pro-inflammatory cytokine production
	STAT3	Inactivation → chronic colitis, Th1 response, reduced IL-10 signaling
	STAT6	Polarization of M2-like macrophages
Intestinal epithelial cells	STAT1	activates antibacterial/anti-inflammatory IDO-1
	STAT3	IL-22-dependent promotion of goblet cells, mucus layer and IEC restitution IL-24-mediated mucus production IL-11-promoted maintenance of mucosal integrity
	STAT5	Deficiency → increased colitis susceptibility, enhanced IEC apoptosis Critical for regeneration of crypt epithelium
	STAT6	IL-13-dependent apoptosis

## Abbreviations

The following abbreviations are used in this manuscript:

CAC: colitis-associated cancer
CCL: chemokine (C-C motif) ligand
CD: Crohn’s disease
CRC: colorectal carcinoma
DC: dendritic cell
GM-CSF: granulocyte macrophage colony-stimulating factor
IBD: inflammatory bowel disease
IEC: intestinal epithelial cell
IFN: interferon
IL: interleukin
JAK: janus kinase
LPMC: lamina propria mononuclear cell
R: receptor
SOCS: suppressor of cytokine signaling
STAT: signal transducer and activator of transcription
TGF-β: transforming growth factor β
TNF-α: tumor necrosis factor α
Th cell: T helper cell
Treg cell: regulatory T cell
UC: ulcerative colitis
VEGF: vascular endothelial growth factor
